# Preferences of patients with depression for medication management: a discrete choice experiment

**DOI:** 10.3389/fpsyt.2025.1626653

**Published:** 2025-09-03

**Authors:** Peng Xie, SiZhu Guo, Hao Yang, YanFen Wang, WenWen Ming, HuangYan Li, Hui-qin Li

**Affiliations:** ^1^ People’s Hospital of Deyang City, Deyang, Sichuan, China; ^2^ Southwest Jiaotong University, The Third Peoples Hospital of Chengdu, Chengdu, Sichuan, China; ^3^ West China Hospital, Sichuan University, Chengdu, Sichuan, China; ^4^ West China School of Nursing, Sichuan University, Chengdu, Sichuan, China; ^5^ School of Nursing, Tongji Medical College, Huazhong University of Science and Technology, Wuhan, Hubei, China

**Keywords:** depressive disorder, depression, adherence, preference, discrete choice experiment, medication management, DCE

## Abstract

**Background:**

Depression threatens people’s health and imposes heavy economic burden on society. Oral antidepressants are the first-line treatment for depression. However, poor adherence in depressed patients contributes to poor clinical outcomes. Effective medication management can improve patient adherence, but the current approaches to medication management in depression have shown limited success. Understanding patients’ needs and preferences can help improve their medication adherence. There are no data on the preferences of depressed patients in developing countries.

**Objective:**

A discrete choice experiment (DCE) was conducted to elicit and quantify the preferences of people with depression for medication management, and to provide references for the development of effective medication management strategies to improve the medication adherence of people with depression.

**Methods:**

The design principles of discrete choice experiments were used to develop the survey instrument. Attributes included adverse reactions, provider, follow-up frequency, cost, follow-up method, and convenience of purchase. A mixed logit model was used to estimate the preferences, willingness to pay (WTP), subgroup analyses based on relapse, and the uptake rates of different medication management program using the NLCOM command.

**Results:**

The preferences of 373 people with depression were analyzed. The six attributes included in this study had a significant impact on preferences of people with depression (P < 0.05). Slight adverse reactions were the most important attribute level (coefficient =0.905), with the highest willingness to pay, and increased program uptake by 0.362. Patients who did not experience recurrence preferred to go to the pharmacy to purchase antidepressants on site. In terms of follow-up methods, those with recurrence experience preferred remote follow-up. Providing face-to-face and telephone/we-chat follow-up by psychiatrists, and with slight adverse reactions, the probability of receiving medication management program increased by 0.478, which was close to the optimal medication management program.

**Conclusion:**

The formulation of medication management strategies should be rooted in the preferences of people with depression. The impact of recurrent depression experiences on preferences should be considered when forming collaborative care teams consisting of psychiatrists, psychiatric nurses, and family physicians to address the complex and multifaceted needs of people with depression.

## 1 Introduction

Depression afflicts approximately 350 million people worldwide and is one of the most common mental disorders (1). The risk of people with depression suffering from type II diabetes, metabolic syndrome and hypertension is significantly increased (2–4). Depression is a major risk factor for suicidal behavior (5, 6) and a major disease in patients with suicide attempts (2). Compared to the general population, the life expectancy of patients with depression is shortened (6). At the same time, depression brings a huge economic burden to society (7, 8). It causes an estimated US $925 billion in lost productivity globally each year and is one of the most expensive diseases (3, 9). It is expected that by 2030, depression will become the top disease burden globally (8). Reducing the growing burden of mental disorders such as depression is therefore a global health priority (9).

The first-line treatment for depression is oral antidepressants (10), and patient compliance is the key to successful treatment of depression with oral antidepressants (9). However, 50% to 70% of patients are poor adherent (11–13). Poor medication adherence leads to increased levels of depression (14), increased recurrence, and increased emergency department visits and hospitalizations (11, 15), resulting in limited depression treatment and failure in depression management (16), and affecting overall health care utilization (17). It is urgent to take corresponding measures to improve the medication adherence of patients with depression.

A study has shown that effective medication management has great potential to improve patient medication adherence (18). However, current medication management strategies for depression are less successful and do not improve patient adherence (19–21). Patient participation in decision-making is considered as an ethical requirement (22), and it is essential to consider patients’ needs and preferences to develop and optimize medication management strategies to improve their medication adherence (19, 23). Incorporating patient preferences into clinical decision making can improve uptake rates, reduce withdrawal during treatment, and optimize treatment adherence (3, 24, 25). Multiple guidelines mention that the treatment of patients with mood disorders should be based on their preferences (26, 27). At the same time, for patients with depression, identifying their preferences by having them weigh the benefits and risks associated with depression treatment can help improve medication adherence (28).

The Discrete Choice Experiment (DCE) originated in the field of economics and is capable of eliciting and quantifying individual preferences. Some scholars have conducted DCE to explore the preferences of people with depression for treatment (29–31). However, these studies have the following limitations: 1) These studies focus on the outcome characteristics of medication, while ignoring the impact of factors outside of medication, such as health guidance and follow-up strategies, on medication adherence in patients with depression; and 2) These studies were conducted in developed countries, and there is a lack of data on treatment preferences among individuals with depression in developing countries. There is a huge gap in the treatment of mental disorders between developed and developing countries (32). In low - and middle-income countries, most people with mental disorders do not receive cost-effective treatment (33). On the contrary, in developed countries, the drug subsidy system is well-established, and the difference in personal economic burden is very small (34). Therefore, the results of previous studies are not applicable to developing countries. We conducted a DCE study in China, a developing country, to obtain data on the preferences of patients with depression for medication management, so as to help clinicians and policy makers in developing countries to formulate effective medication management strategies and improve medication adherence of patients with depression.

## 2 Materials and method

### 2.1 Aims

To obtain data on the preferences of patients with depression for medication management by conducting a DCE, so as to help clinicians and policy makers in developing countries to formulate effective medication management strategies and improve medication adherence of patients with depression.

### 2.2 Design

This study followed the design principles of the DCE study, and data collection was conducted from June 2024 to February 2025. Our study adhered to the checklist for reporting guidelines on good practices in health care regarding DCE developed by the International Society for Pharmacoeconomics and Outcomes Research (ISPOR) (35). Our study protocol has been published (36).

### 2.3 Identifying and defining attributes and levels

We determined attributes and levels according to the recommended methods (25, 35). The electronic databases, Wanfang database, Embase, Web of Science, China National Knowledge Infrastructure (CNKI) and PubMed, were used to review the relevant literature on adherence in people with depression. Snowballing method was used to obtain more comprehensive information. Based on this, potential attributes and levels were identified and an interview outline was determined. Subsequently, people with depression were interviewed one-on-one, and data saturation was reached after 15 interviews. Afterwards, focus group discussions were conducted with depressed patients who did not participate in the one-on-one interviews, and a total of 4 focus group discussions (FGDs) were conducted with 3 depressed patients in each group. One-on-one consultations were conducted with 1 health economist, 1 psychiatric nurse, 1 psychiatrist, and 1 pharmacist (all of whom worked for more than ten years) to obtain more attributes and to determine the applicability of potential attributes in current medical health care. Ten possible attributes have been obtained. Some researchers believe that the optimal number of attributes for DCE is 6 (37). Therefore, people with depression were required to vote on the importance of these attributes, ultimately including the six attributes that they consider the most important, namely “adverse reactions” “provider” “follow-up frequency” “cost” “follow-up method” and “ convenience of purchase”. See Attachment A for attributes and levels details (36).

### 2.4 Generating choice sets and designing questionnaires

A unlabeled design was used to construct the choice sets to avoid the attention of respondents to the target attributes (38). A fractional factorial design was performed using Ngene software to generate 36 choice sets, and they were randomly divided into 3 blocks to reduce the cognitive burden on responders. Each choice set included an “opt-out” option to avoid overestimation of participation rates (39). To examine the consistency of respondent selection, the second choice set for each block was repeatedly included as the thirteenth-choice set.

The survey questionnaire consists of three parts. The first part introduces the purpose, content, questionnaire filling requirements, and precautions of the research. The second part was a general information questionnaire used to collect the socio-demographic characteristics of the respondents. The third part is the DCE selection task. In this part, each attribute and its levels are described in detail, and an example of choice set is given for “warm-up”. Attachment B shows an example of choice set (36).

Twenty patients with depression were invited to participate in the pilot test, and the statements of the questionnaire were modestly adjusted to improve clarity and intelligibility based on the results and feedback from the pilot test.

### 2.5 Participants and data collection

The subjects of this study were depressed patients without other psychiatric disorders. Patients who could understand and fill out the questionnaire, has the experience of taking antidepressants currently or in the past six months, and are willing to participate in the survey were included.

Convenience sampling was adopted to determine the respondents. Data collection includes both online and on-site methods. Emails or We-chat accounts of patients who met the inclusion criteria were obtained from the admission registries of four hospitals in Sichuan Province, and questionnaires were provided to them via We-chat or email. At the same time, patients who meet the inclusion criteria are invited from the outpatient and inpatient departments of these hospitals to participate in the survey, and questionnaires are collected on-site. The data collection period is from June 2024 to February 2025.

### 2.6 Data analysis

Stata16.0 software was used to analyze the data. Descriptive statistics were performed on the socio-demographic characteristics of the respondents. The preferences of people with depression were estimated using a mixed logit model. All coefficients in the model are assumed to be normally distributed. The cost attribute was modeled as a continuous variable to estimate the willingness to pay (WTP) of patients with depression, and the remaining attribute levels were coded as dummy variables. The value of the coefficient β indicates the relative importance of the attribute level to the reference level in the medication management scheme, while the statistical significance of the coefficient β indicates whether the attribute level influences the selection of depressed patients. Grouping based on sociodemographic variables to explore the preference differences among people with depression with different demographic characteristics. The nlcom command is used to simulate the uptake rate, which is the change in the probability of depressed patients receiving medication management programs when one or more attribute levels change compared to the baseline medication management program. For all calculations, statistical significance was indicated at a two-sided p < 0.05 level.

## 3 Results

### 3.1 Characteristics of respondents

A total of 500 patients with depression from four hospitals in Sichuan province were invited, of which 420 patients agreed to participate in the survey, and 411 patients completed the questionnaire. Among the 411 completed questionnaires, 38 participants (9.2%) were excluded due to failing the consistency test, resulting in 373 participants included in the final analysis. Among them, there were more females (n=218) than males (n=155) (accounting for 58.45% and 41.55% respectively). Most patients were aged 18 – 29 years (124 respondents, accounting for 33.24%). The number of patients with recurrent depression is 197, accounting for 52.82%. Patients with high school education account for 37.73%, and the highest number of respondents with monthly income between 2000 - 6000, accounting for 47.99%. Most of the respondents (69.97%) took multiple antidepressants. Details are provided in **Table 1**.

**Table 1:** Characteristics of respondents.

Characteristics	Respondents (N = 373)	
	N	%
Gender		
Male	155	41.55
Female	218	58.45
Age, years		
18-29	124	33.24
30-39	97	26.01
40-49	73	19.57
50-59	49	13.14
60-69	21	5.63
≥70	9	2.41
Highest level of education		
Primary school and below	16	4.68
Junior high school	201	58.77
Senior high school	68	19.88
College degree and above	57	16.67
Relapse experiences		
Yes	197	52.82
No	176	47.18
Monthly income		
<2000	25	6.71
2000-6000	179	47.99
6000-10000	98	26.27
≥10000	71	19.03
Types of medication		
single	112	30.03
multiple	261	69.97
Occupational status		
Working full-time	123	32.98
Working part-time	94	25.20
Working casual	49	13.14
Not working	41	10.99
Home duties and/or caring responsibilities	29	7.77
Retired	37	9.92
Area of residence		
Metro/city	191	51.21
Regional	113	30.29
Rural	69	18.50

### 3.2 Overall results

The results of the mixed logit estimates for the total sample are reported in **Table 2**. The WTP measures how much respondents are willing to pay to improve the characteristics of the medication management program, and the results are reported in **Table 3**. We found that the coefficients at all attribute levels were significant, that is, p < 0.05, indicating that the six attributes included in this study are important for adherence to antidepressant medication in depressed patients. The results showed that the coefficient of the cost attribute was negative, suggesting that depressed patients preferred the less costly medication management option. The most salient finding was that adverse reaction profiles overwhelmingly determined patients’ preferences. The most important attribute for patients with depression is the adverse reactions of antidepressants. Their preference coefficients for slight and mild adverse reactions are 0.905 (95% CI 0.6629873 - 1.146418) and 0.832 (95% CI 0.591335 - 1.073632), respectively. Patients demonstrated the highest WTP to avoid severe side effects, valuing slight adverse reactions at 1,517.77 CNY (95% CI: 712.37 - 2,323.18) and mild reactions at 1,396.62 CNY (95% CI: 786.14 - 2,007.09) annually. This substantial WTP differential - exceeding all other attributes by 3 – 4 fold - underscores that tolerability should be the primary consideration when prescribing antidepressants. Follow-up modality emerged as the second most critical factor influencing adherence. Patients strongly preferred blended care models combining face-to-face and remote follow-ups (coefficient=0.777, WTP = 1,304.25 CNY) over telemedicine-only approaches. Psychiatrists were the most preferred follow-up providers (coefficient=0.513, 95%CI 0.3193968 - 0.7071995). It is worth noting that psychiatric nurses were also preferred by people with depression as follow-up providers (coefficient=0.232, 95%CI 0.3193968 - 0.7071995). 96%CI 0.0220205 - 0.4414176). Although medication accessibility showed statistical significance (coefficient=0.196), its relatively modest WTP (328.51 CNY) and wider confidence intervals (-6.56 to 663.59 CNY) position it as a secondary consideration compared to side effects and follow-up quality. This indicates that while patients demonstrated a positive preference for this attribute, they were not willing to pay a statistically significant additional amount for it. At the same time, the statistics of the SD coefficients for all attribute levels are significant, suggesting that there is heterogeneity in the preference of people with depression for attribute levels.

**Table 2:** Mixed logit estimates for total sample.

Attribute levels (reference level)	Coefficient (s.e)	95%CI		SD (s.e)	95% CI	
cost	-0.0005961** (0.0001557)	-0.0009013	-0.0002909			
Provider (Pharmacist)						
Psychiatrist	0.513** (0.099)	0.3193968	0.7071995	1.007** (0.122)	0.766532	1.247783
Psychiatric specialist nurse	0.232** (0.107)	0.0220205	0.4414176	1.210** (0.117)	0.9799441	1.439787
Follow-up frequency (Once every six weeks)						
Once every two weeks	0.391** (0.094)	0.20722	0.574574	0.660** (0.122)	0.4212767	0.8990491
Once every four weeks	0.231* (0.101)	0.0324181	0.4288724	0.640** (0.130)	0.3864861	0.894164
Adverse reactions (Severe)						
Slight	0.905** (0.123)	0.6629873	1.146418	1.560** (0.120)	1.363268	1.835319
Mild	0.832** (0.123)	0.591335	1.073632	1.082** (0.118)	0.8496185	1.313511
Follow-up methods (Telephone or We-chat)						
Face to face	0.670** (0.102)	0.4697493	0.8704229	0.401* (0.193)	0.0213631	0.7800373
Alternate between face-to-face & telephone/We-chat	0.777** (0.118)	0.5457901	1.009068	0.849** (0.117)	0.6201725	1.078064
Convenience of purchase (Generally convenient)						
Very convenient	0.196* (0.085)	-0.0288383	0.3627983	1.042** (0.088)	0.8697558	1.21443

*p<0.05, **p<0.01.

**Table 3:** Willingness to pay for attribute levels.

Attribute levels	WTP (CNY)	95%CI	
Provider-psychiatrist	861.13406	310.9588	1411.3093
Provider- psychiatric specialist nurse	388.74319	21.098733	756.38765
Follow-up Frequency- once every two weeks	655.78793	288.427	1023.1489
Follow-up Frequency- once every four weeks	386.94173	54.08438	719.79907
Adverse Reactions-slight	1517.7738	712.36587	2323.1816
Adverse Reactions-mild	1396.6152	786.13998	2007.0905
Follow-up Methods-face to face	1124.1692	517.68806	1730.6503
Follow-up Methods-alternate between face-to-face & telephone/We-chat	1304.2533	654.29538	1954.2112
Convenience of Purchase-very convenient	328.51437	-6.560001	663.58875

### 3.3 Results of subgroup analyses

Subgroup analysis stratified by depression recurrence status revealed clinically distinct preference patterns, and the results are shown in **Table 4**. For people with depression who have not experienced recurrence, the three most important attribute levels are, in turn, adverse reactions–slight (coefficient=1.386), follow-up methods-alternate between face-to-face & telephone/We-chat (coefficient=1.363), and adverse reactions-mild (coefficient=1.046). In addition, their preference for convenience of purchase-general convenience was negative (coefficient=-0.267), indicating that they prefer to purchase antidepressants on-site at pharmacies. For patients with recurrent depression, the three most important attribute levels are, in turn, adverse reactions-slight (coefficient=0.748), follow-up frequency-once every four weeks (coefficient=0.699), and adverse reactions-mild (coefficient=0.620). It is worth noting that compared with telephone/WeChat follow-up method, patients with recurrent depression have no significant preference for face-to-face and telephone/We-chat alternate, while their preference for attribute level face-to-face is negative (coefficient=-0.462), which indicates that they prefer remote follow-up, that is, follow-up through telephone/We-chat.

**Table 4:** Results of subgroup analysis.

Attribute levels (reference level)	Patients without relapse experiences		Patients with relapse experiences	
	Coefficient (s.e)	SD (s.e)	Coefficient (s.e)	SD (s.e)
Cost	-0.0013715**(0.0002313)		-0.0008043** (0.0002097)	
Provider (Pharmacist)				
Psychiatrist	1.016**(0.173)	0.997**(0.184)	0.482**(0.123)	0.803**(0.185)
Psychiatric specialist nurse	0.757**(0.175)	0.986**(0.137)	0.176(0.148)	1.180**(0.168)
Follow-up frequency (Once every six weeks)				
Once every two weeks	0.870**(0.149)	0.666**(0.180)	0.536**(0.138)	0.948**(0.148)
Once every four weeks	0.515**(0.145)	0.588*(0.182)	0.699**(0.141)	0.977**(0.155)
Adverse reactions (Severe)				
Slight	1.386**(0.283)	1.739**(0.189)	0.748**(0.212)	1.067**(0.181)
Mild	1.046**(0.300)	1.183**(0.173)	0.620**(0.167)	1.530**(0.162)
Follow-up methods (Telephone or We-chat)				
Face to face	0.881**(0.199)	0.118(0.393)	-0.462*(0.216)	0.883**(0.201)
Alternate between face-to-face & telephone/We-chat	1.363**(0.269)	0.534(0.278)	0.161(0.133)	0.262(0.387)
Convenience of purchase (Generally convenient)				
Very convenient	-0.267*(0.133)	0.592**(0.161)	0.367*(0.113)	0.859**(0.147)

*p<0.05, **p<0.01.

### 3.4 Simulated medication management preferences with changes in program characteristics

**Figure 1:**
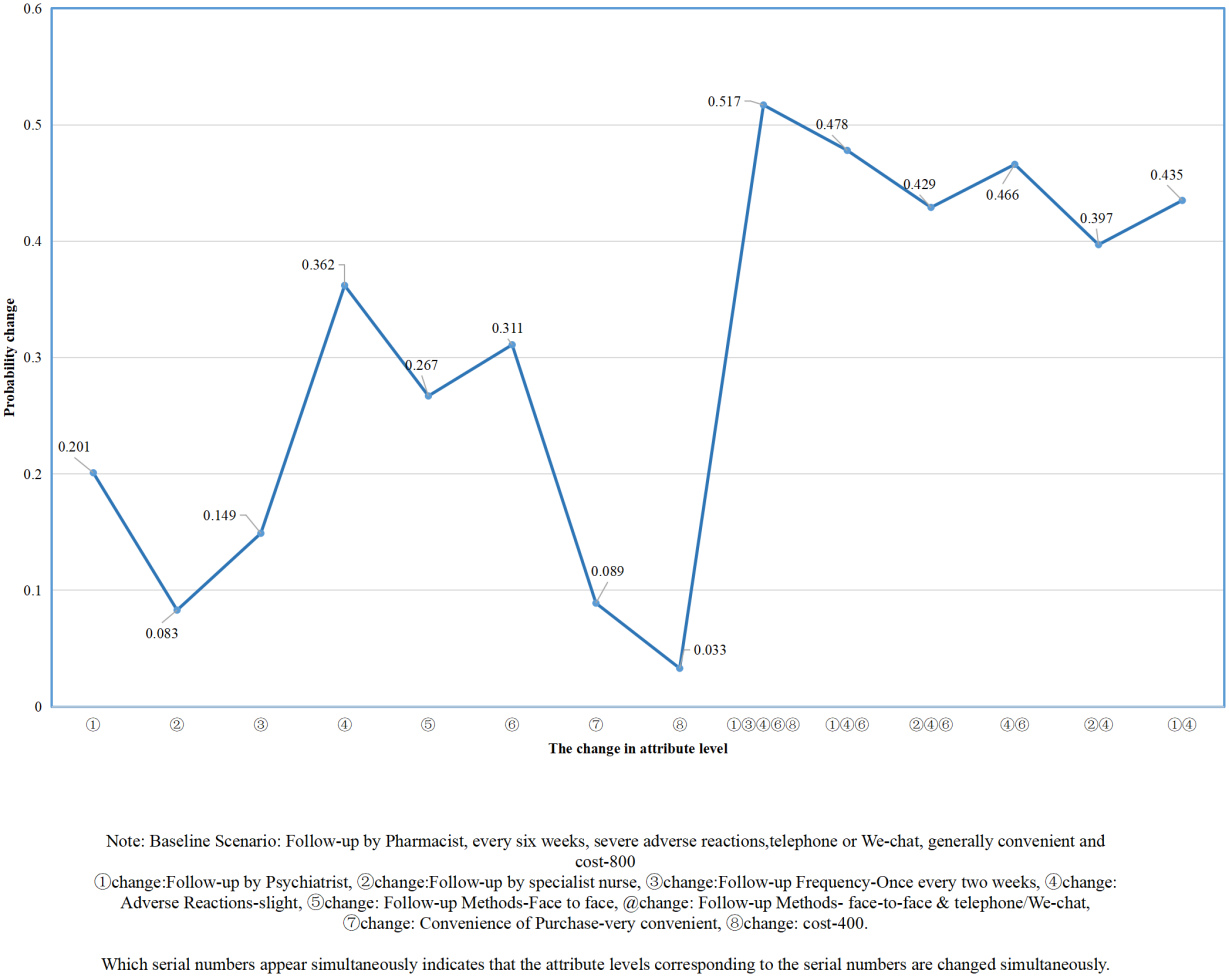
Simulated medication management preferences with changes in program characteristics.

The probability of people with depression receiving a medication management program after a change in one or more attribute levels is simulated, and the main findings are reported in **Figure 1**. When a single attribute level changes, adverse reactions have the greatest impact on preferences of people with depression. When adverse reactions change from severe to slight and from severe to mild, the probability of people with depression receiving medication management program increases by 0.362 and 0.333, respectively. When the follow-up method changed from telephone/we-chat to face-to-face and telephone/we-chat alternately, the acceptance probability of medication management program of patients with depression increased by 0.311. When multiple attribute levels change simultaneously, the optimal medication management program can increase the probability of people with depression accepting the program by 0.517. The optimal program should meet the following requirements: (1) psychiatrists as follow-up providers; (2)follow-up once every two weeks; (3) slight adverse reactions; (4)follow-up methods were face-to-face and telephone/we-chat alternately; (5) it is very convenient to purchase drugs; and (6) it costs 400 yuan per month. When changing fewer attribute levels, such as face-to-face and telephone/we-chat follow-up provided by psychiatrists and slight adverse reactions, the probability of accepting the medication management program increased by 0.478, which was close to the optimal medication management program.

## 4 Discussion

To the best of our knowledge, this is the first DCE conducted in a developing country to explore the preferences of people with depression for medication management. Our study included factors other than medication (such as follow-up methods and follow-up frequency), which made up for the limitation of previous studies that focused on the outcome characteristics of medication and ignored the influence of other factors on patients’ medication adherence. Our study found that patients with depression attach great importance to adverse reactions and follow-up methods, and they are willing to pay more money for less adverse reactions and more diverse follow-up methods. Psychiatrists are the most preferred follow-up providers for patients with depression, followed by psychiatric nurses. The changes in attribute levels affect the uptake rate of medication management program by patients with depression. The change from severe to slight adverse reactions is the single factor that has the greatest impact on the uptake rate. The optimal medication management program had the greatest impact on uptake rates when multiple attribute levels were changed simultaneously. However, similar results can be achieved by changing fewer attribute levels.

The most preferred attribute level for patients with depression is slight adverse reactions, and they have the highest WTP for this attribute level. A study has shown that the most common reason for poor adherence in people with depression is concern about long-term effects and possible adverse reactions of medications (40). Weight gain, sexual dysfunction, nausea and vomiting, and headaches are common adverse reactions related to antidepressants, with a prevalence rate of 74.3% in patients with depression (41). The chronic torment of adverse drug reactions makes people with depression eager to get rid of this pain, which may be why they value slight adverse reactions the most. The efficacy of second-generation antidepressants is similar to that of first-generation antidepressants, and second-generation antidepressants have low side effects and already play an important role in the treatment of depression (42, 43). However, in low-income and middle-income countries, first generation antidepressants are still widely used, which may further exacerbate the occurrence of adverse drug reactions (44). At the same time, a study has shown that adverse drug reactions are significantly associated with polypharmacy (41, 45). Rational use of drugs requires that patients have access to a medication appropriate for their medical condition over a period of time, at a dose appropriate for their specific needs, and at the lowest feasible cost to the patients and their community (46), which means that the use of several (usually five or more) medications per day may not always be clinically necessary. Therefore, controlling the number of drugs and prioritizing the prescription of second-generation antidepressants may be an effective way to reduce adverse drug reactions in the treatment of patients with depression.

Our study showed that face-to-face and telephone/we-chat alternately were the most preferred follow-up methods for patients with depression. Depressed patients are afraid of being stigmatized due to their depression (47, 48), which may lead them to refuse antidepressant treatment and follow-up. In China, the follow-up and treatment of patients with depression are mainly conducted face-to-face in hospital outpatient departments, and patients with depression are usually lack of motivation due to disease, which makes the participation in outpatient follow-up low. China currently has high telephone coverage and 1.13 billion we-chat users. Alternately face-to-face and telephone/we-chat follow-up is more convenient and more in line with patients’ expectations.

In our study, the non-significant WTP for purchase convenience despite significant preference may suggest that: (1) patients view convenience as a basic expectation rather than a premium feature worth paying extra for, (2) the perceived value of convenience is highly variable across respondents leading to wide confidence intervals, or (3) convenience may be valued more in terms of time savings rather than monetary terms.

The quality of medical follow-up is a key aspect of depression treatment (49). Our findings showed that the most preferred follow-up provider for patients with depression was a psychiatrist, followed by a psychiatric nurse. There is a severe shortage of psychiatrists in China, and community-based mental health services are underdeveloped, resulting in uneven distribution of psychiatrists and many patients being unable to access mental health services (50–52). Therefore, specialized psychiatric nurses seem to be the optimal providers of medication management. A greater role for specialist nurses can improve care delivery and control costs (53). At the same time, nurse-led primary care can improve the quality of life and obtain better patient satisfaction (54). Therefore, training more psychiatric specialist nurses to play a greater role in the medication management of patients with depression is a more economical option.

Subgroup analysis results show that patients with depression who have not experienced recurrence are more inclined to purchase medication on-site at pharmacies. In recent years, there have been multiple incidents of selling inferior and fake drugs in China (55), which has raised public doubts about the quality of medicines. The quality of medicines can be checked by purchasing medicines on site. This may be the reason for the preferences of patients with first-episode depression to purchase medicines at the pharmacy/hospital site. Drug sales points should be established to meet the preferences of depressed patients who have experienced their first episode or no recurrence, and to improve their convenience in purchasing drugs at physical pharmacies.

Patients with depression who have experienced relapse expect a less frequent follow-up, which is consistent with a previous study in which depressed patients preferred a consultation frequency of once every two weeks compared to once a week (56). Meanwhile, people with depression with recurrence experience prefer remote follow-up, that is, follow-up by telephone/we-chat. The recurrence rate of depression is high, with nearly 80% of patients experiencing a recurrence of depression within 5 years (57). In addition, it has been shown that 15% of patients with depression exhibit a chronic course (58). Although face-to-face follow-up may obtain more comprehensive drug-related knowledge, the experience of disease recurrence and longer course of disease improve patients’ disease coping experience and drug-related knowledge, so they may choose a more convenient follow-up method, namely remote follow-up. Patients with depression have higher compliance to remote intervention (59). In the medication management of people with depression, providing remote follow-up for patients who have experienced recurrence of depression may improve their medication adherence.

When multiple attribute levels change at the same time, fewer attribute levels change have a greater impact on the choice of medication management program for patients with depression. When other characteristics remained unchanged, adverse reactions were slight and services were provided by psychiatrists alternately through face-to-face or phone/we-chat, the probability of receiving medication management program increased by 0.478, which was close to the optimal medication management program. These findings suggest two clear implementation pathways:

For well-resourced settings, adopting the full optimal program can maximize treatment acceptance. For resource-constrained clinics, focusing on the core triad of provider type, follow-up method, and side effect profile can yield substantial benefits.

The consistent pattern across analyses indicates that while comprehensive programs produce the best outcomes, strategic improvements in key modifiable factors can meaningfully enhance treatment acceptance in diverse clinical settings. Although psychiatrists are considered important as providers of medication management, the manpower for mental health care is generally neglected in middle-income and low-income countries. In the case of China, there is a severe psychiatric workforce shortage, which is further exacerbated by high turnover rates. In terms of cost, specialized nurses seem to be a more economical choice. Family doctors play a crucial role in treating mental disorders in primary healthcare (60). People who are socially and economically disadvantaged are unlikely to receive regular sources of care from family doctors (61), which limits the accessibility of medication management provided by doctors. Collaborative care may have provided better health outcomes for patients and provided higher quality treatment for depression (62, 63). Therefore, specialized training of nurses and formation of an assisted care team composed of psychiatrists, psychiatric nurses, and family doctors to address the complex and multifaceted individual needs of people with depression in a diverse follow-up method may be more effective medication management strategies in the future.

### 4.1 Limitations

This study has some limitations. Firstly, although we recruited patients from four hospitals in Sichuan Province to reduce sample bias, this may not fully represent the views of people with depression in other regions of China. Secondly, our use of convenience sampling, while logistically efficient, may have resulted in a sample that does not fully capture the diversity of adherence behaviors, particularly among high-risk or hard-to-reach populations. Caution is therefore warranted when extrapolating these findings to broader clinical settings. Thirdly, although we combined online and on-site survey responses to ensure sample diversity, we did not perform a formal sensitivity analysis to compare preferences between these subgroups. Differences in unobserved characteristics (e.g., digital literacy, socioeconomic status) may introduce bias. Future studies could stratify recruitment to assess mode effects. Finally, in order to reduce the cognitive burden of the respondents, only the six most important attributes and their levels were included in our study, and the excluded attribute levels may also be important, which limits our discussion of the results. It may be the research direction of future scholars to include more comprehensive attribute levels and carry out national large sample surveys.

### 4.2 Clinical implications and practical significance

Our findings provide actionable guidance for clinical practice to enhance medication adherence in depression. The strong patient preference for minimal adverse effects (with a 36.2% increase in program uptake for slight side effects) underscores the need for clinicians to prioritize well-tolerated antidepressants and proactively address side effect concerns through patient education and dose adjustments. The differential follow-up preferences based on relapse history—remote options (telephone/WeChat) for patients with prior relapse versus in-person pharmacy visits for non-relapsed patients—highlight the importance of personalized care pathways.

The near-optimal strategy combining psychiatrist-led follow-up (face-to-face or telephone) with low-side-effect regimens (47.8% uptake increase) offers a practical, evidence-based model for implementation. These results are particularly relevant for healthcare systems aiming to optimize resource allocation, as they demonstrate patients’ willingness to pay for better treatment experiences. By adopting these tailored approaches—including telehealth integration for high-risk patients and collaborative care models—clinicians may improve adherence rates and, consequently, treatment outcomes in real-world settings. This is especially valuable in resource-limited areas where improving adherence may reduce the economic burden of ineffective depression treatment.

## 5 Conclusion

In our study, the characteristics of medication management that were considered important for people with depression reflected the expectations of them for health management services. The findings of this study provide references for the development and optimization of medication management strategies. The development of medication management strategies should be rooted in the preferences of people with depression. The impact of recurrent depression experiences on preferences should be considered when forming an collaborative care team consisting of psychiatrists, psychiatric nurses, and family doctors to address the complex and multifaceted individual needs of depressed patients.

## Data Availability

The original contributions presented in the study are included in the article/supplementary material. Further inquiries can be directed to the corresponding author.
